# Supplementary material for: Silicon and glass very large scale microfluidic droplet integration for terascale generation of polymer microparticles
Source: Nat Commun. 2018 Mar 26;9:1222. doi: 10.1038/s41467-018-03515-2 (PMC5964316; doi:10.1038/s41467-018-03515-2)
Supplement: Supplementary file 1 — Supplementary Information(PDF 4323 kb) [file 41467_2018_3515_MOESM1_ESM.pdf]

ELECTRONIC SUPPLEMENTARY INFORMATION

Silicon and Glass Very Large Scale Microfluidic Droplet Integration for Terascale Generation of Polymer Microparticles

Sagar Yadavali[1, *], Heon-Ho Jeong [2†], Daeyeon Lee [2], David Issadore [1,2,3,*]

[1]Department of Bioengineering, University of Pennsylvania, Philadelphia, Pennsylvania 19104, USA

[2]Department of Chemical and Biomolecular Engineering, University of Pennsylvania, Philadelphia, Pennsylvania 19104, USA

[3]Electrical and Systems Engineering, School of Engineering and Applied Sciences, University of Pennsylvania, Philadelphia, Pennsylvania 19104, USA

[†]Current address: Department of Chemical and Biomolecular Engineering, Chonnam National University, Yeosu, Jeonnam, 59626, Republic of Korea

Sagar Yadavali (yadavali@seas.upenn.edu), David Issadore (issadore@seas.upenn.edu)

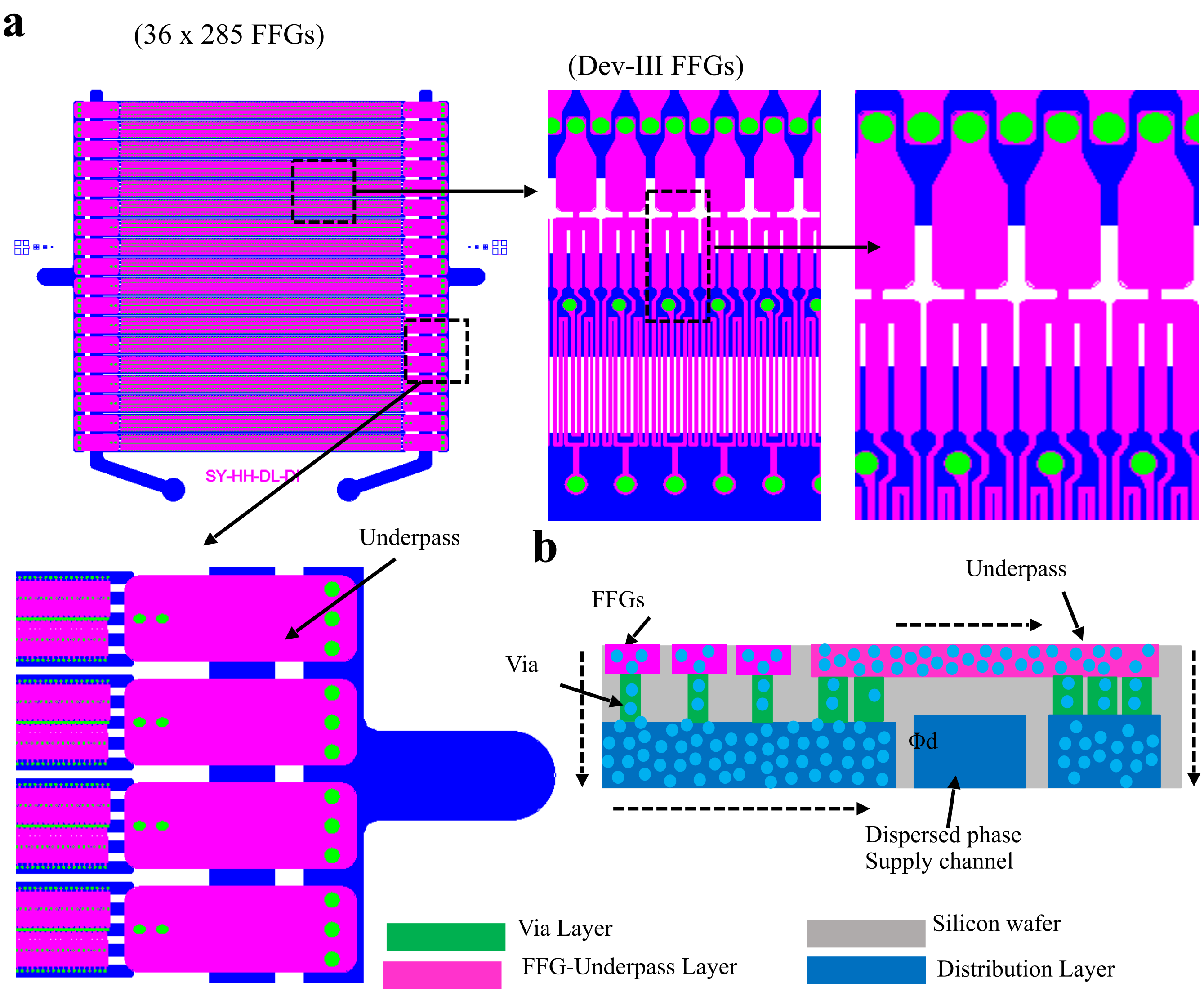

Supplementary Figure 1: Schematic layer diagram of VLSDI showing different layers. (a) The device contains 36 rows by 285 columns with a total of 10,260 FFGs (b) Schematic shows the cross section of an underpass channels and the direction of droplets movement from FFGs to the outlet channel.

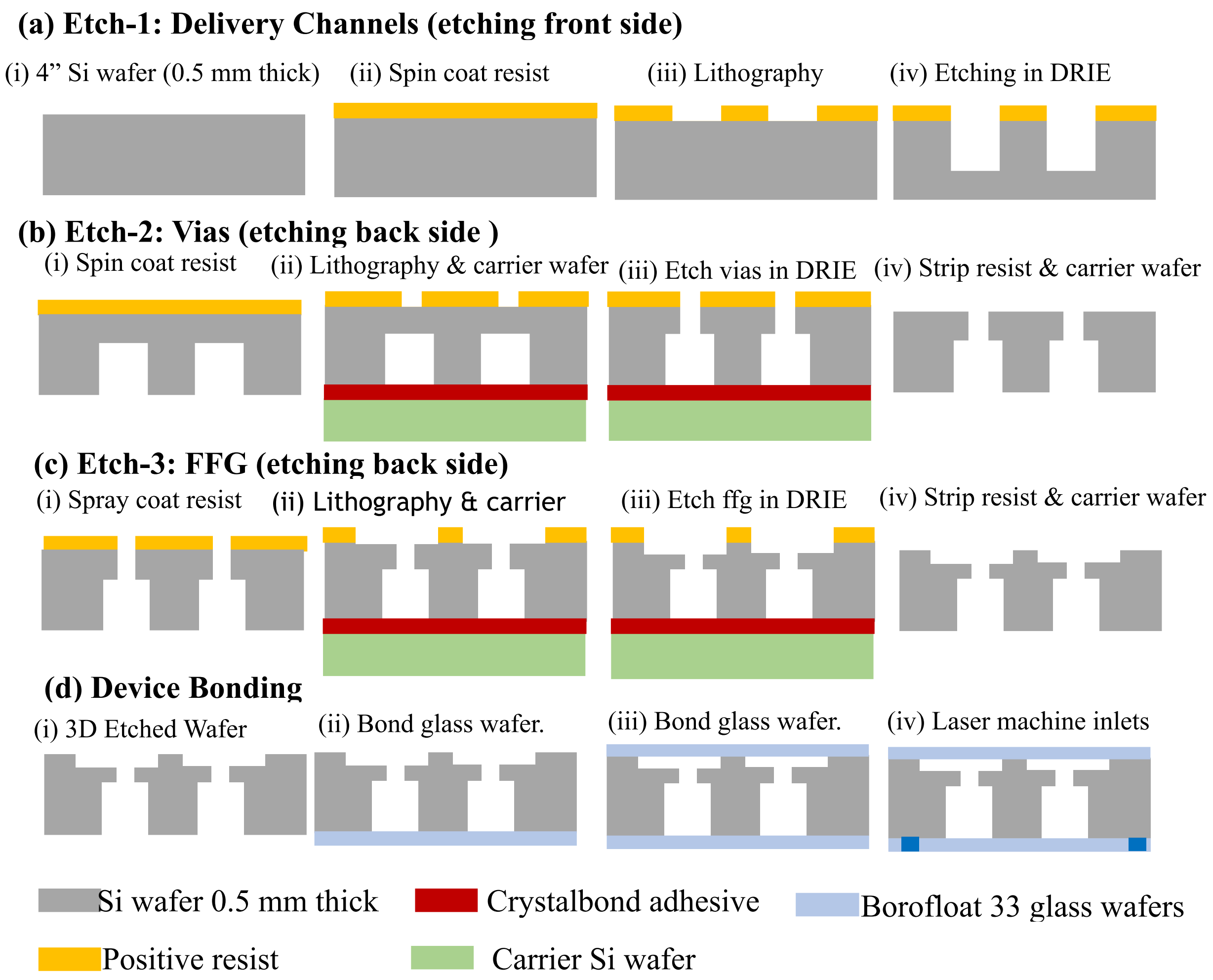

Supplementary Figure 2: 3D etching of Silicon wafer for Dev I, Dev II and Dev III VLSDI chips. **(a)** Etching of silicon wafer on the front side for delivery channels. Positive resist SPR 220.7 with 16 μm thick is coated and patterned for delivery channels. The wafer is then etched 380 μm deep in deep reactive ion etching (DRIE) and then the resist is stripped off. **(b)** The wafer is flipped and 12 μm resist is coated on the wafer and patterned for vias. The wafer is then bonded to another Silicon wafer using crystal bond at 65C for through silicon via etching. The crystal bond wafer is then removed by placing it on the hotplate at 65C, remaining crystal bond to the wafer is removed by immersing in acetone. **(c)** The wafer is spray coated with 4 μm resist and patterned for flow focusing generators (FFGS). The wafer is then crystal bonded to another Silicon wafer. The wafer is etched for desired depth. **(d)** The wafers are then bonded to glass wafers on both sides by anodic bonding. Excimer laser is used to punch holes into the device for inlets and outlets collections.

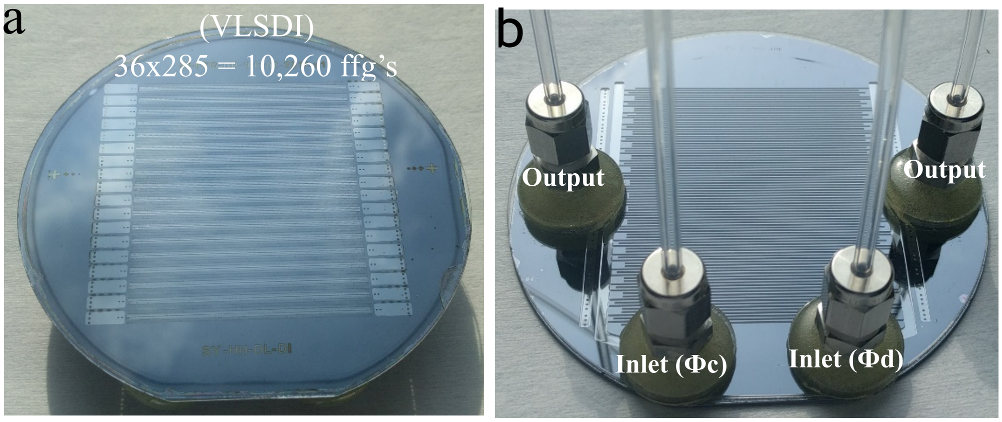

Supplementary Figure 3: (a) Top view of VLSDI chip, that shows FFGs and underpass channels. (b) Backside view of the device that shows supply and delivery channels.

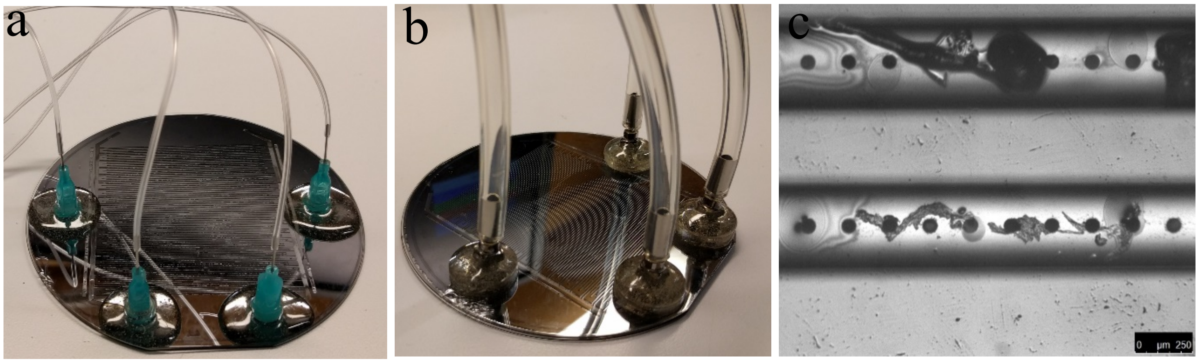

Supplementary Figure 4: (a-c) Syringe tip connections using compressed tygon tubing and direct tube fittings resulted debri in the device delivery channels over time.

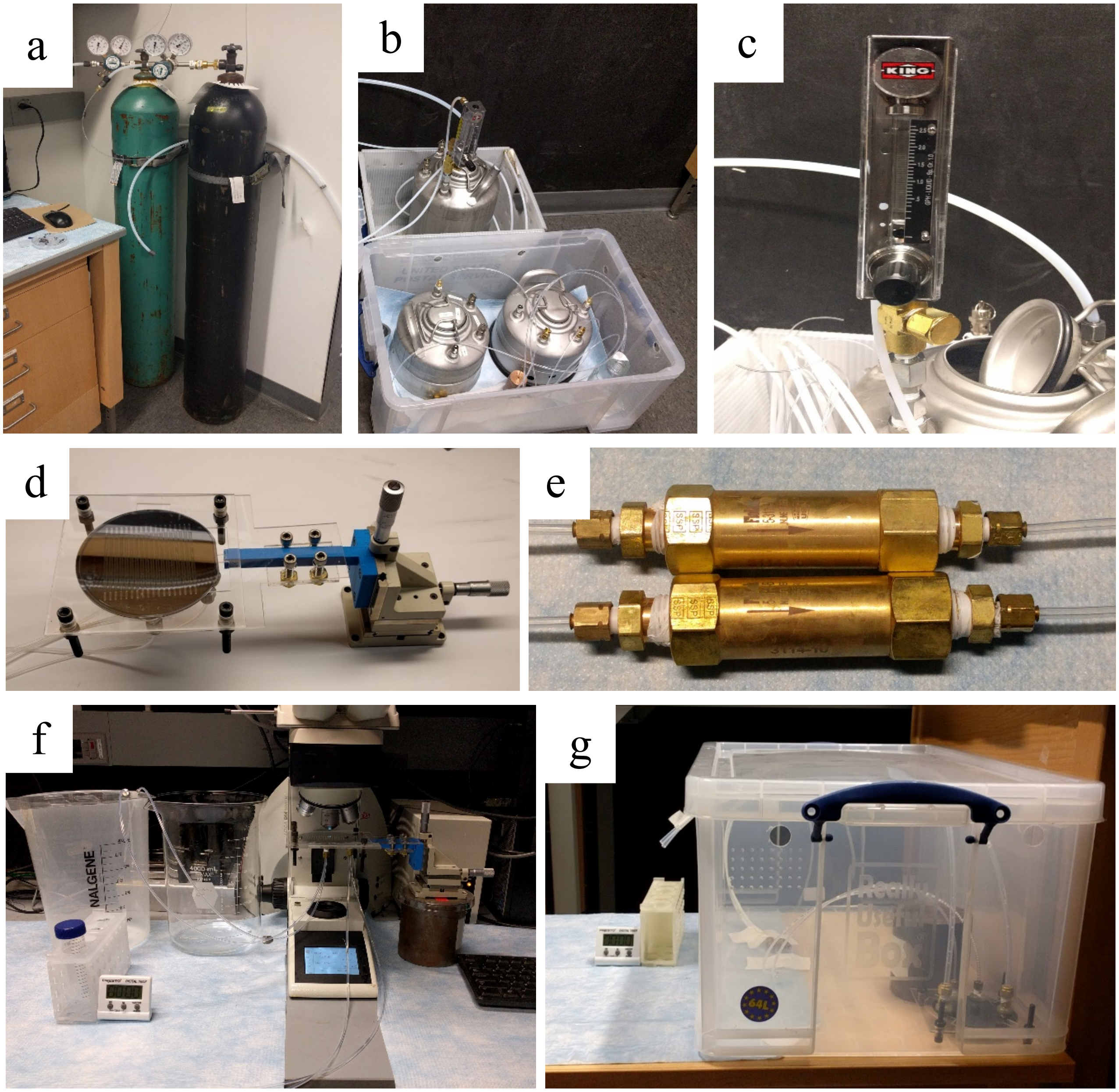

Supplementary Figure 5: Detailed experimental setup used to test VLSDI chips. (a) Pressured tanks are used for pressure driven flow for continuous and dispersed phase fluids. **(b)** 1 and 3 gallon pressure vessels are used for dispersed and continuous phase fluids. **(c)** An inline flowmeter is used to measure the flow rate of continuous phase fluid (Water). **(d)** An acrylic housing is built to house the VLSDI chip and mounted on to a xyz stage to move the chip. **(e)** Inline filters are placed in the inlet tubing's of both dispersed and continuous flow fluids to filter out debri and particulates. **(f)** Experimental setup to test the device and observe the droplet generation in microscope. **(g)** VLSDI chip is housed in a larger 64 L container for high pressure experiments.

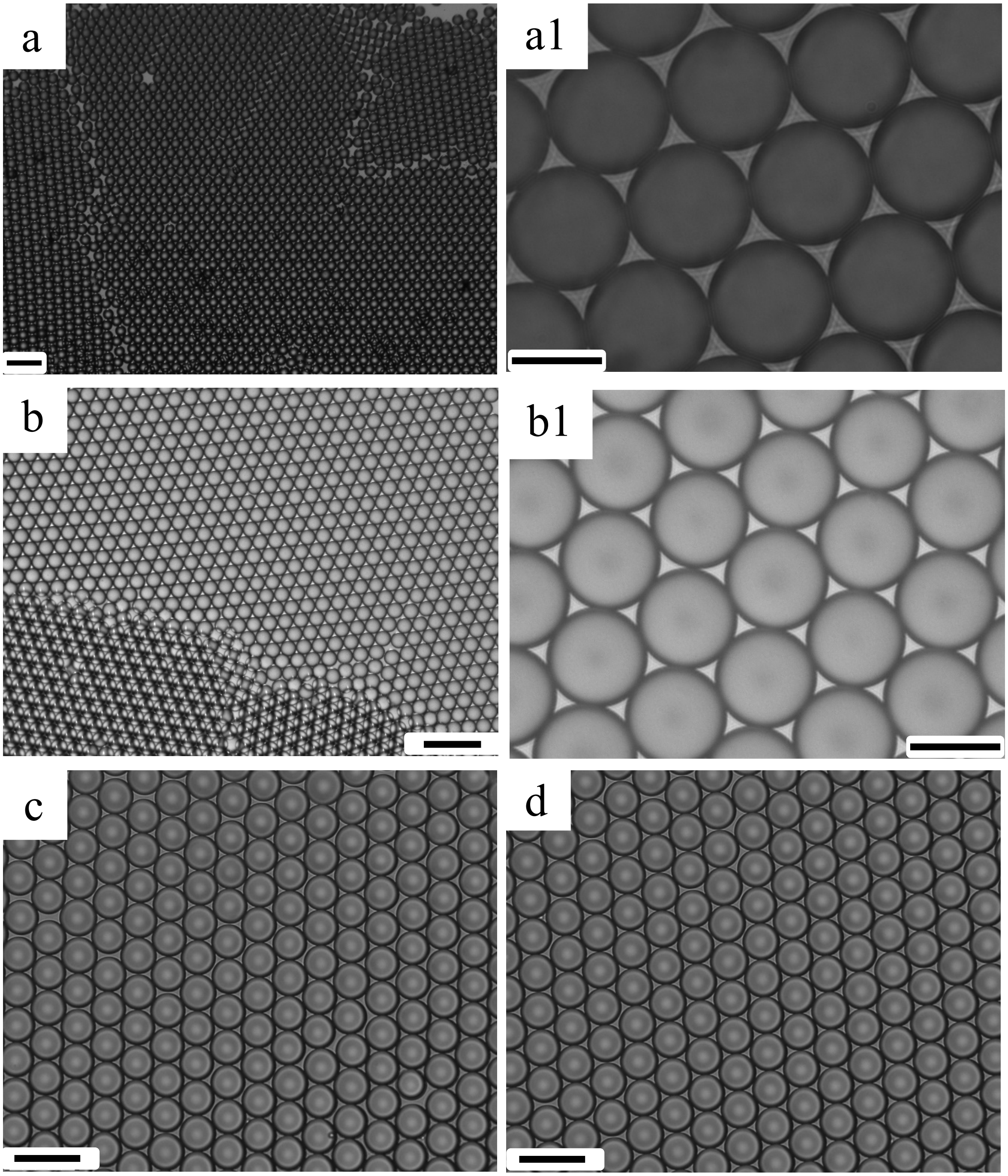

Supplementary Figure 6: High throughput generation of hexadecane droplets generated from Dev III VLSDI. Dispersed phase (Φ_d) is hexadecane and continuous phase (Φ_c) is 2wt% Tween80 in water. **(a-a1)** Droplets generated for Φ_d=1.20 Lhr^{-1} and Φ_c = 1.40 Lhr^{-1} (D= 37.5 µm, CV = 1.75%). Scale bar : 100 µm and 25 µm. **(b-b1)** Droplets generated for Φ_d = 4.00 Lhr^{-1} and Φ_c = 6.25 Lhr^{-1} (D = 28.5 µm, CV = 2.92%). Scale bar : 100 µm and 25 µm. **(c)** Droplets generated for Φ_d=0.40 Lhr^{-1} and Φ_c = 5.60 Lhr^{-1} (D = 25.5 µm, CV = 2.30%). Scale bar 50 µm. **(d)** Droplets generated for Φ_d =1.00 Lhr^{-1} and Φc=2.80 Lhr^{-1} (D= 31.6 µm,

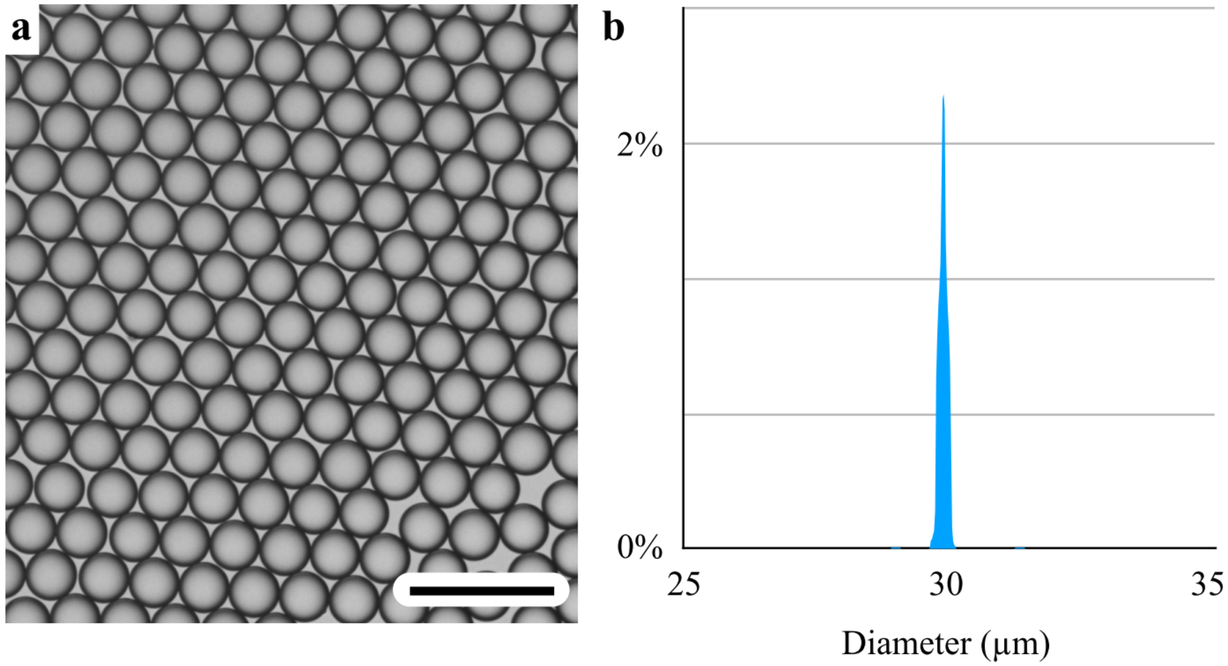

Supplementary Figure 7: High throughput generation of a viscous dispersed phase from Dev III VLSDI. Dispersed phase (Φ_d) is Mineral Oil (viscosity 30 cps) and continuous phase (Φ_c) is 2wt% Tween80 in water. The dispersed phase was $\Phi_d = 2.2$ Lhr^{-1} and continuous phase flow rate was $\Phi_c = 11.5$ Lhr^{-1}. (a) A micrograph of the droplets. The scale bar is 75 μm. A histogram of $n = 500$ droplets. The droplets had a mean diameter of 24.3 μm and a coefficient of variation CV = 2.3%.
