# Supplementary material for: Silicon and glass very large scale microfluidic droplet integration for terascale generation of polymer microparticles
Source: Nat Commun. 2018 Mar 26;9:1222. doi: 10.1038/s41467-018-03515-2 (PMC5964316; doi:10.1038/s41467-018-03515-2)
Supplement: Supplementary file 3 — Description of Additional Supplementary Files(DOCX 15 kb) [file 41467_2018_3515_MOESM3_ESM.docx]

**Description of Additional Supplementary Files**

File Name: Supplementary Movie 1

Description: Movie shows the generation of oil in water (O/W) droplets at different positions in Dev III VLSDI. Oil phase: Hexadecane, Water phase: Deionized water with 2 wt% Tween 80.

File Name: Supplementary Movie 2

Description: Movie shows the generation of oil in water (O/W) droplets as a function of flow rates in Dev III VLSDI. Oil phase: Hexadecane, Water phase: Deionized water with 2 wt% Tween 80.

File Name: Supplementary Movie 3

Description: Movie shows the calibration of emulsion throughput for oil in water (O/W) droplets as a function of flowrates in Dev III VLSDI. Oil phase: Hexadecane, Water phase: Deionized water with 2wt% Tween 80.

File Name: Supplementary Movie 4

Description: Movie shows the generation of oil in water (O/W) droplets at different positions in Dev III VLSDI. Oil phase: Mineral oil (viscosity 30 cps), Water phase: Deionized water with 2 wt% Tween 80.

File Name: Supplementary Movie 5

Description: Movie shows the generation of oil in water (O/W) droplets at different positions in VLSDI-TJN. Oil phase: Hexadecane, Water phase: Deionized water with 2wt% Tween 80.

File Name: Supplementary Movie 6

Description: Movie shows the generation of emulsion templates at different positions in Dev III VLSDI. The movie also shows dichloromethane evaporation from templates, polymer precipitation and the flowrate of emulsion generation. Oil phase: Dichloromethane with 10 wt% (133 g/L) of polycaprolactone. Water phase: Deionized water with 2 wt % of PVA. (Φd = 0.82 L/hr, Φc = 4.08 L/hr)

File Name: Supplementary Movie 7

Description: Movie shows a live video for the generation of emulsion templates at maximum production of polycaprolactone (277 grams/hour). (Φd = 2.09 L/ hr, Φc = 5.11 L/hr)
